# Preclinical study of CC223 as a potential anti-ovarian cancer agent

**DOI:** 10.18632/oncotarget.17753

**Published:** 2017-05-10

**Authors:** Zhenzhen Jin, Huanfu Niu, Xuenan Wang, Lei Zhang, Qin Wang, Aijun Yang

**Affiliations:** ^1^ Center for Reproductive Medicine, Affiliated Hospital of Jining Medical University, Jining, China; ^2^ Department of Pathology and Laboratory Medicine, Clinical Microarray Core, David Geffen School of Medicine, University of California Los Angeles, Los Angeles, California, USA

**Keywords:** ovarian cancer, mTOR, CC223, signaling

## Abstract

Aberrant activation of mTOR contributes to ovarian cancer progression. CC223 is a novel and potent mTOR kinase inhibitor. The current study tested its activity against human ovarian cancer cells. We showed that CC223, at nM concentrations, inhibited survival and proliferation of established/primary human ovarian cancer cells. Further, significant apoptosis activation was observed in CC223-treated ovarian cancer cells. CC223 disrupted assembly of mTOR complex 1 (mTORC1) and mTORC2 in SKOV3 cells. Meanwhile, activation of mTORC1 and mTORC2 was almost completely blocked by CC223. Intriguingly, restoring mTOR activation by introduction of a constitutively-active Akt1 only partially inhibited CC223-induced cytotoxicity in SKOV3 cells. Further studies showed that CC223 inhibited sphingosine kinase 1 (SphK1) activity and induced reactive oxygen species (ROS) production in SKOV3 cells. At last, oral administration of CC223 potently inhibited SKOV3 xenografted tumor growth in nude mice. The results of this study imply that CC223 could be further studied as a potential anti-ovarian cancer agent.

## INTRODUCTION

Ovarian cancer is one of the leading cancers among women [1–4]. The incidence of this devastating malignancy has been rising [3, 4]. The current treatment options of ovarian cancer, including the combination of surgery and platinum-based chemotherapy, are very limited [1, 2]. Further, it is estimated that two-third of newly-diagnosed ovarian cancers are advanced diseases, which are remarkably resistant to current treatment [5–7].

Mammalian target of rapamycin (mTOR) is the central player of the phosphatidylinositol 3-kinase (PI3K)-Akt-mTOR cascade, which is vital for malignant transformation and cancer progression [8–12]. PI3K-Akt-mTOR cascade is often over-expressed and/or over-activated in human ovarian cancer, especially in clear cell carcinoma and endometrioid adenocarcinoma [13]. mTOR over-activation is associated with a number of cancerous behaviors, including cell cycle progression, cell survival, growth and proliferation, as well as metabolism, motility, angiogenesis, apoptosis-resistance, and genomic instability [8–12]. Therefore, mTOR represents potentially important therapeutic target for ovarian cancer [14, 15].

Growing evidences have identified at least two multi-protein mTOR complexes: mTOR complex 1 (mTORC1) and mTOR complex 2 (mTORC2) [16, 17]. Rapamycin and its analogs (*i.e*. RAD001, CCI-779) are mTORC1 inhibitors, which bind to FKBP12 to partially inhibit mTORC1 (but not mTORC2) [8]. Very recent studies have developed CC223 as a novel, potent, selective, and orally bioavailable mTOR kinase inhibitor [18–20]. It blocks mTOR kinase activity, therefore presumably shutting of both mTORC1 and mTORC2 [18–20]. The preclinical study tested CC223's activity against human ovarian cancer cells both *in vitro* and *in vivo*.

## RESULTS

### The effect of CC223 to ovarian cancer cell survival and proliferation

**Figure 1:**
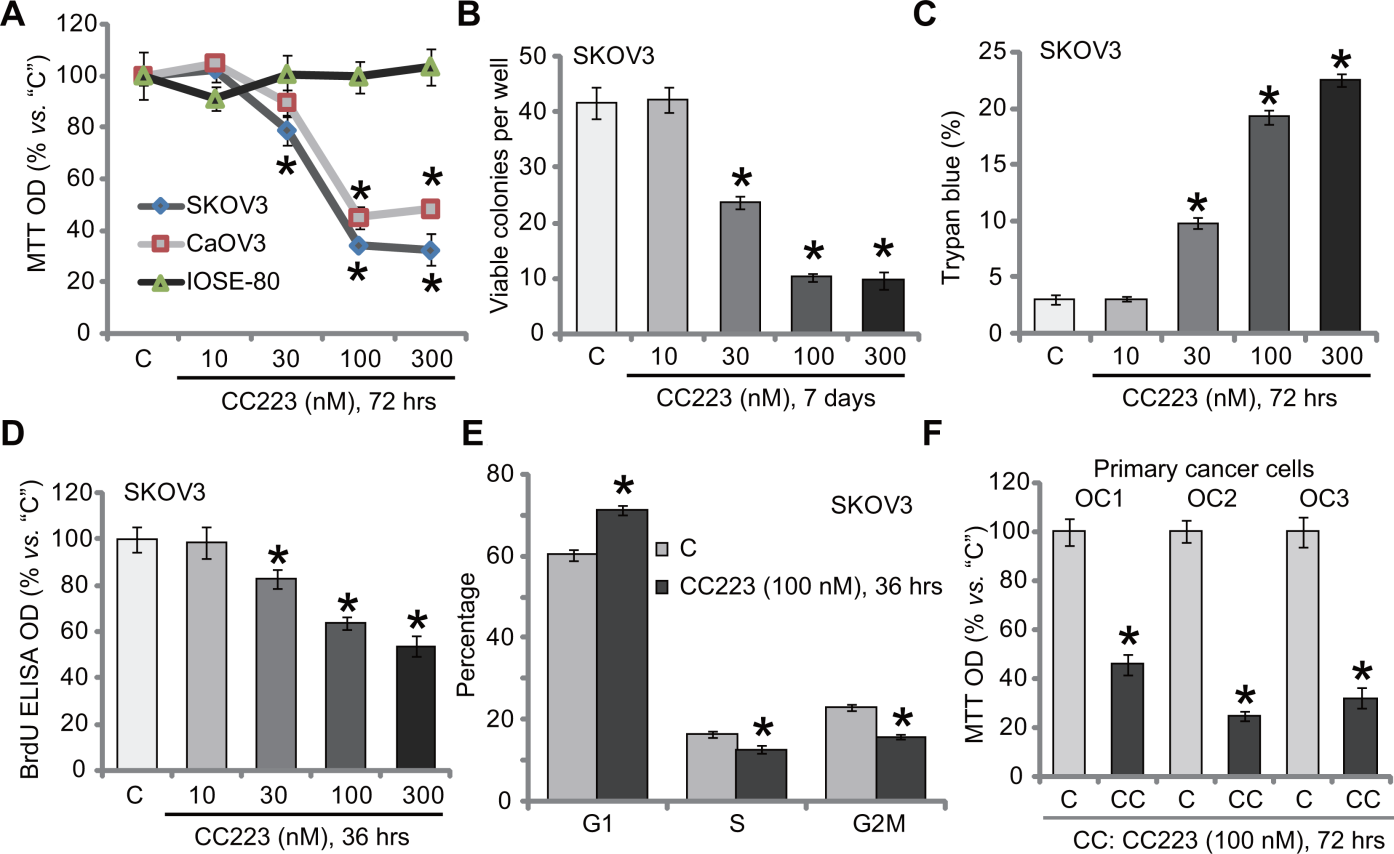
The effect of CC223 to ovarian cancer cell survival and proliferation Ovarian cancer cell lines (SKOV3 and CaOV3), the ovarian surface epithelial (IOSE-80) cells or the patient-derived primary ovarian cancer cells (“OC1”, “OC2” and “OC3” lines) were either left untreated (“C”, same for all Figures), or treated with CC223, cells were further cultured in the CC223 conditional medium for implied time; Cell survival/death (**A**–**C**, **F**), proliferation (**D**) and cell cycle progression (**E**) were tested by the listed assays. Data were presented as mean ± standard deviation (SD, *n* = 5). *p < 0.05 *vs*. “C” group. Results in this figure were repeated three times, similar results were always obtained.

To study the potential activity of CC223 again ovarian cancer, established ovarian cancer cell lines, SKOV3 and CaOV3 [21, 22], were treated with CC223 at various concentrations (10–300 nM). MTT assay results in Figure 1A demonstrated that CC223 inhibited ovarian cancer cell survival, in a concentration-dependent manner. The IC-50s of CC223 for SKOV3 cells and CaOV3 cells were 64.32 ± 5.21 nM and 88.17 ± 6.32 nM, respectively. On the other hand, treatment with CC223 at same concentration (10–300 nM) was yet non-cytotoxic to primary human ovarian surface epithelial (IOSE-80 line) cells [23] (Figure 1A). To further study the activity of CC223 in ovarian cancer cells, clonogenicity assay was performed. Treatment with CC223 (30–300 nM) potently decreased the number of viable SKOV3 colonies (Figure 1B). Further studies showed that the percentage of trypan blue positive (“dead”) SKOV3 cells was significantly increased after CC223 (30–300 nM) treatment (Figure 1C). These results suggested that CC223 was cytotoxic to cultured human ovarian cancer cells.

Next, SKOV3 cell proliferation was also analyzed. BrdU assay results showed that CC223 dose-dependently inhibited SKOV3 cell proliferation, indicated by decreased BrdU ELISA OD (Figure 1D). Importantly, treatment with CC223 (100 nM) in SKOV3 cells induced increase of G1 phase cells, but decrease of S and G2-M phase SKOV3 cells, indicating G1-S arrest (Figure 1E). These results imply that CC223 inhibited SKOV3 cell proliferation. To study CC223's activity in primary human cancer cells, a total three lines of patient-derived primary ovarian cancer cells were established, which were named as “OC1, OC2 and OC3”. Results in Figure 1F confirmed that CC223 (100 nM, 72 hours) significantly decreased survival (or MTT OD) of these primary cancer cells. CC223's sensitivity in these primary cancer cells was slightly different (Figure 1F). Collectively, these results show that CC223 inhibits human ovarian cancer cell survival and proliferation.

### The effect of CC223 on ovarian cancer cell apoptosis

**Figure 2:**
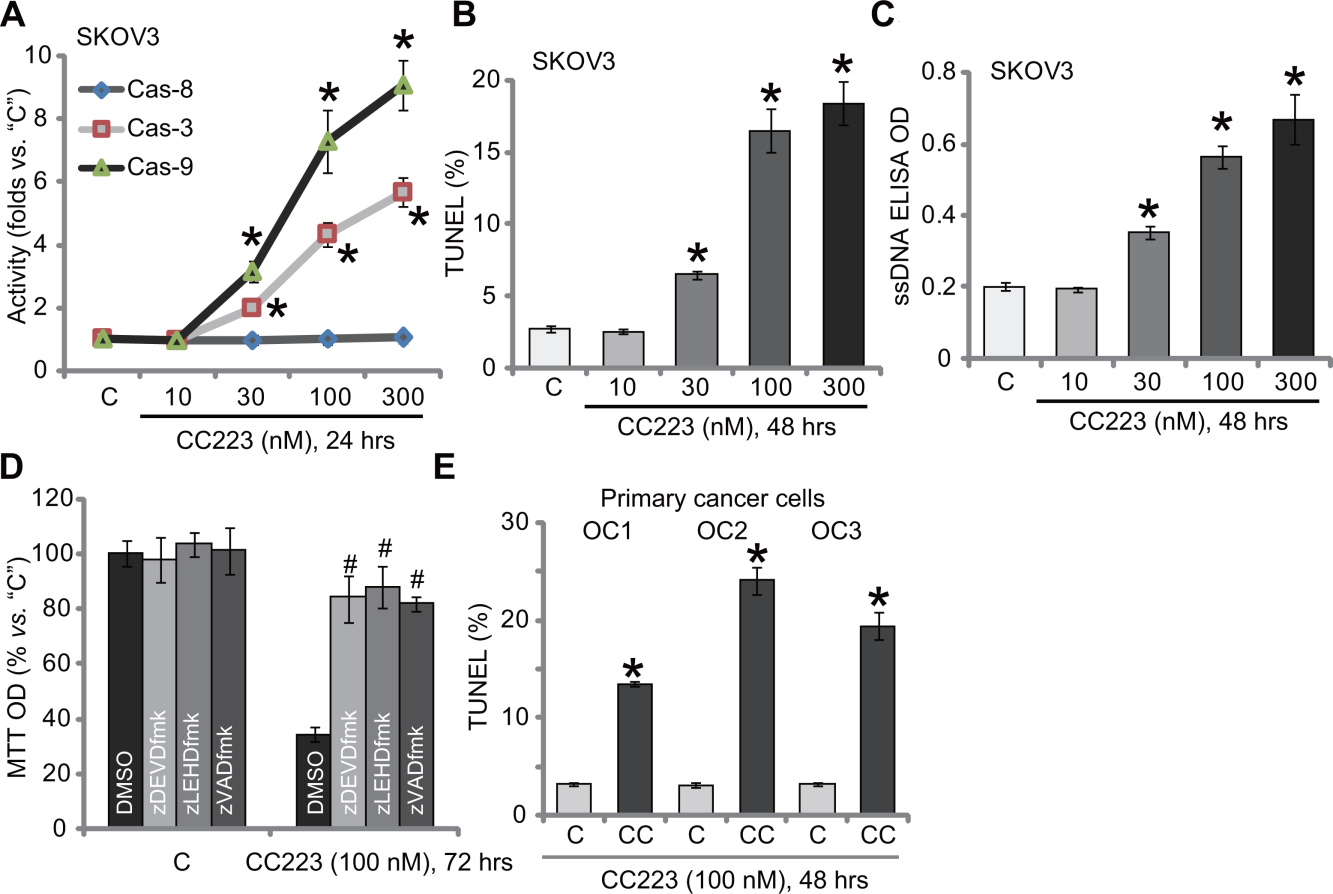
The effect of CC223 on ovarian cancer cell apoptosis SKOV3 cells or the patient-derived primary ovarian cancer cells (“OC1”, “OC2” and “OC3” lines) were treated with CC223 at indicated concentration, cells were further cultured; Cell apoptosis was tested by the listed assays (**A**–**C**, **E**); For (**D**), SKOV3 cells were also pre-treated for 1 hour with 50 μM of caspase inhibitor z-DEVD-fmk, z-LEHD-fmk or z-VAD-fmk, cell survival was tested by MTT assay. Data were presented as mean ± standard deviation (SD, *n* = 5). *p < 0.05 *vs*. “C” group.^#^ *p* < 0.05 *vs*. CC223 only (D). Results in this figure were repeated three times, similar results were always obtained.

Cell apoptosis was also tested. Caspase activity assay results in Figure 2A demonstrated that CC223 dose-dependently activated caspase-3 and caspase-9 in SKOV3 cells, indicating activation of endogenous apoptosis pathway [24, 25]. On the other hand, the activity of caspase-8, indicator of exogenous apoptosis pathway [24, 25], was not changed before and after CC223 treatment (Figure 2A). Further studies showed that CC223 (30–300 nM) treatment in SKOV3 cells dramatically increased the number of TUNEL positive cells (Figure 2B) and ssDNA ELISA OD (Figure 2C), again confirming apoptosis activation. Caspase apoptosis inhibitors were applied next. MTT assay results showed that the caspase-3 inhibitor z-DEVD-fmk, the caspase-9 inhibitor z-LEHD-fmk as well as the pan caspase inhibitor z-VAD-fmk all inhibited CC223 (100 nM)-induced SKOV3 cell viability reduction (Figure 2D). Therefore, CC223 apparently activated caspase-dependent apoptotic cell death. In the primary human ovarian cancer cells, treatment with CC223 (100 nM) similarly induced apoptosis activation, or TUNEL ratio increase (Figure 2E).

### CC223 blocks activation of mTORC1 and mTORC2 in SKOV3 cells

**Figure 3:**
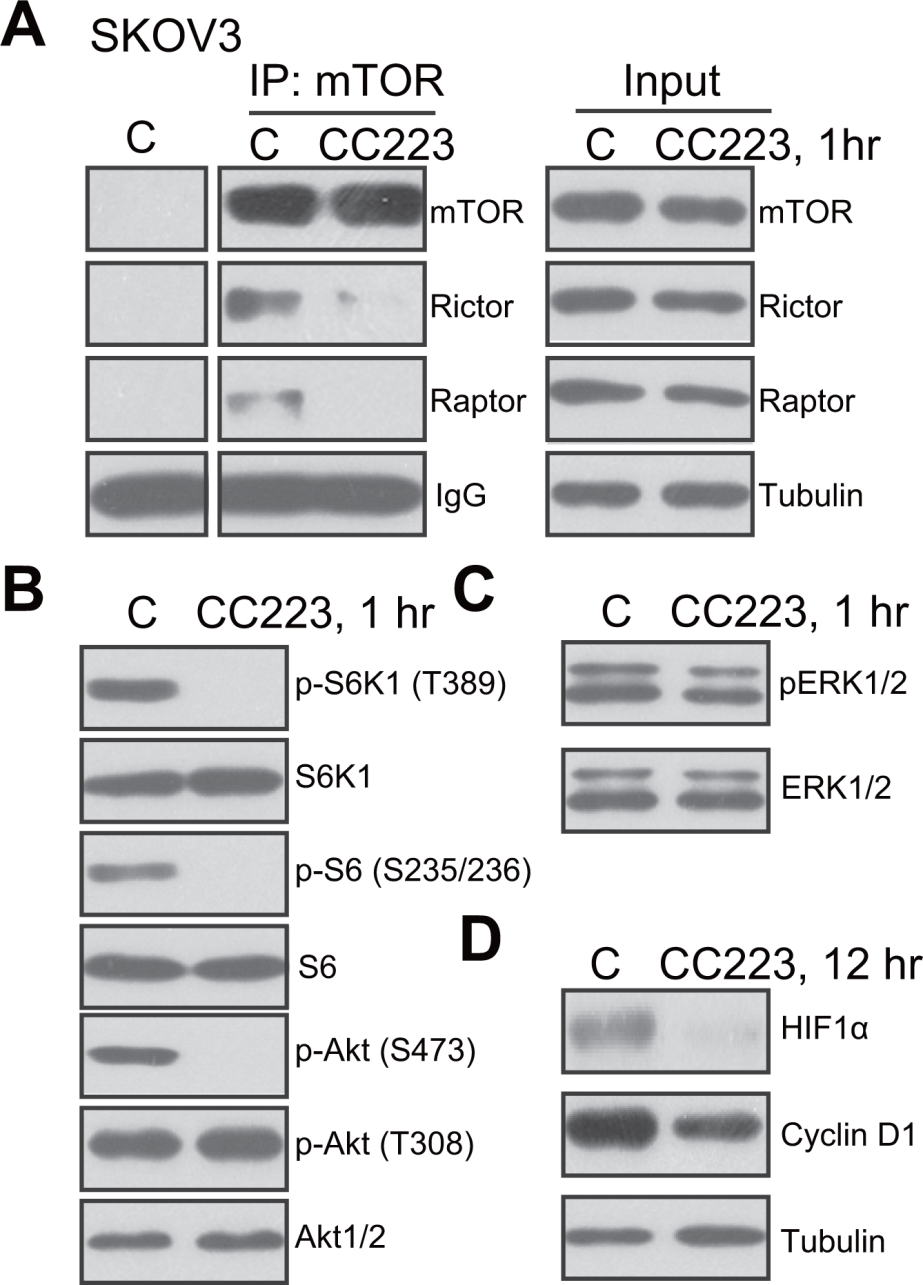
CC223 blocks mTORC1 and mTORC2 activation in SKOV3 cells SKOV3 cells were treated with CC223 (100 nM), cells were further cultured for implied time, mTOR-Raptor and mTOR-Rictor assembly was tested by Co-immunoprecipitation (“Co-IP”) assay (**A**) left panel, “IP”); Expressions of listed proteins were tested by the Western blotting assay (A, “Input” and (**B-D**). Results in this figure were repeated three times, similar results were always obtained.

CC223 is a recently-developed mTOR kinase inhibitor [18–20], its effect on mTOR activation was tested. Co-immunoprecipitation (“Co-IP”) assay was applied to test the integrity of mTOR complexes. Results showed that treatment with CC223 (100 nM, 1 hour) in SKOV3 cells disrupted assembly of both mTOR-Raptor (mTORC1 complex [26]) and mTOR-Rictor (mTORC2 complex [27]) (Figure 3A). Expressions of above-mentioned mTOR complex proteins were not inhibited by the CC223 treatment (Figure 3A, “Input”). Further studies showed that CC223 treatment in SKOV3 cells almost completely blocked phosphorylation (“p-”) of S6K1 and S6, both are mTORC1 indicators (Figure 3B). p-Akt Ser473, the indicator of mTORC2 activation, was also largely inhibited after CC223 treatment (Figure 3B). On the other hand, p-Akt Thr-308 (Figure 3B) and p-ERK1/2 (Figure 3C) were not changed by CC223 treatment. Expressions of above-mentioned total kinases were not decreased by CC223 (Figure 3B and 3C). Notably, two well-known mTOR-dependent proteins, including cyclin D1 and hypoxia-inducible factor-1α (HIF1α) [28–30], were also downregulated after CC223 treatment in SKOV3 cells (Figure 3D). These results together confirm that CC223 blocks mTORC1 and mTORC2 activation in SKOV3 cells.

### Restoring mTOR activation only partially inhibited CC223-induced cytotoxicity in SKOV3 cells

**Figure 4:**
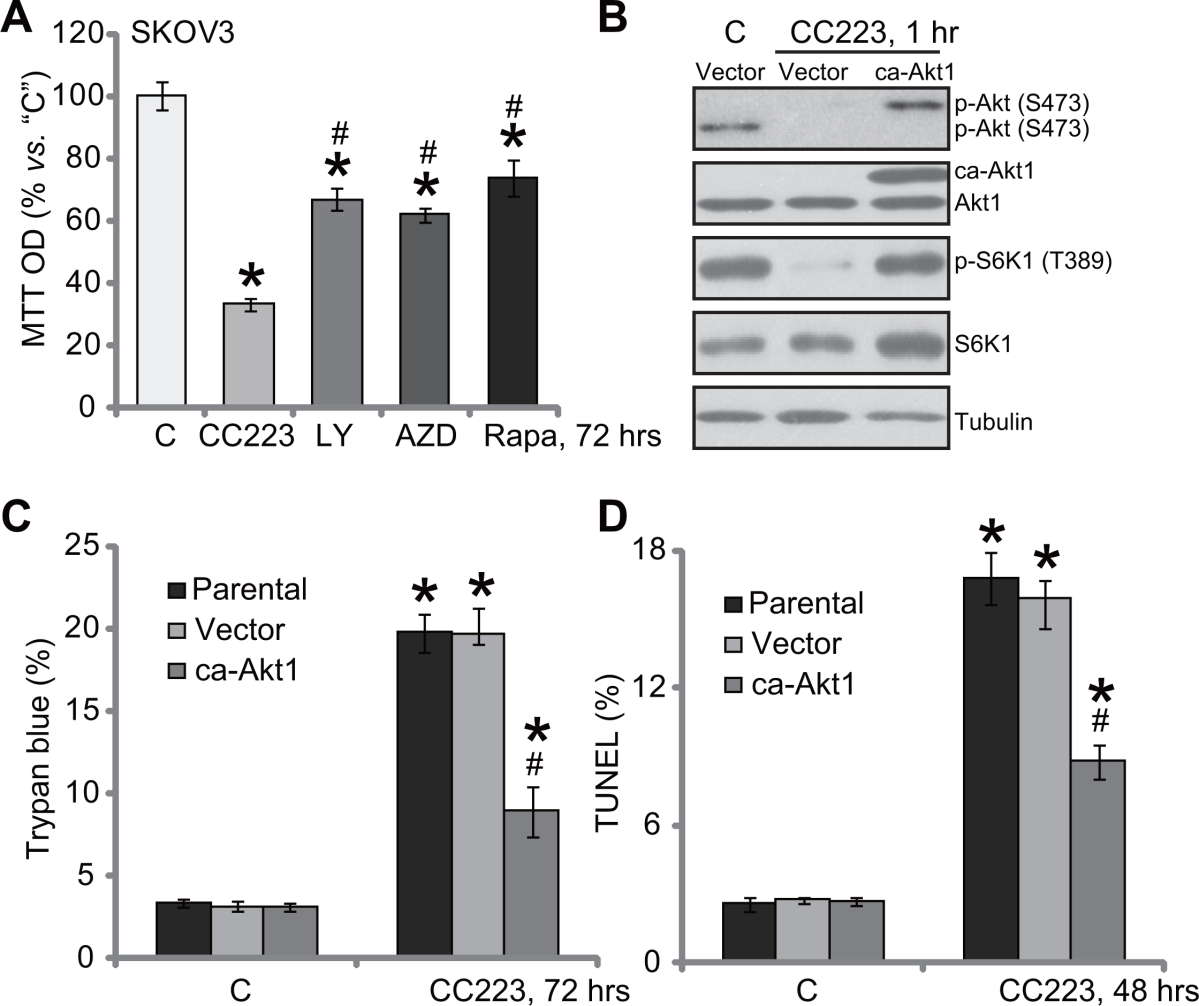
Restoring mTOR activation only partially inhibited CC223-induced cytotoxicity in SKOV3 cells SKOV3 cells were treated with 100 nM CC223, LY294002 (“LY”), AZD-2014 (“AZD”) or rapamycin (“Rap”) for 72 hours; Cell survival was tested by MTT assay (**A**). SKOV3 cells, stably expressing the constitutively-active Akt (“ca-Akt1”) or empty vector, were treated with/out 100 nM of CC223 for indicted time; Expressions of listed proteins were shown (**B**); Cell death (Trypan blue assay, C) and apoptosis (TUNEL assay, D) were tested as well. Data were presented as mean ± standard deviation (SD, *n* = 5). “Parental” stands for parental control SKOV3 cells (C and D). *p < 0.05 *vs*. “C” group. ^#^ *p* < 0.05 *vs*. CC223 (A). ^#^*p* < 0.05 *vs*. CC223 of Vector group (**C** and **D**). Results in this figure were repeated three times, similar results were always obtained.

We compared CC223's activity with other known PI3K-Akt-mTOR inhibitors. MTT assay results in Figure 4A demonstrated that CC223 was significantly more potent in killing SKOV3 cells than same concentration (100 nM) of PI3K-Akt-mTOR inhibitors, including mTORC1 inhibitor rapamycin [31], the other mTOR kinase inhibitor AZD-2014 [32] and the PI3K-Akt-mTOR pan inhibitor LY294002 [33]. These results suggest that CC223 might exert functions other than mTOR inhibition in killing SKOV3 cells. To test this hypothesis, a constitutively-active Akt1 (ca-Akt1) [12, 34] was introduced to SKOV3 cells (Figure 4B). ca-Akt1 completely restored activation of mTORC1 (p-S6K1) and mTORC2 (p-Akt Ser473) in CC223-treated cells (Figure 4B). Yet, ca-Akt1 only partially inhibited (but not reversed) CC223-induced SKOV3 cell death (Figure 4C) and apoptosis (Figure 4D). Therefore these results showed that restoring mTOR activation can only partially inhibit CC223-induced SKOV3 cell death.

### CC223 inhibits SphK1 and induces ROS production in SKOV3 cells

**Figure 5:**
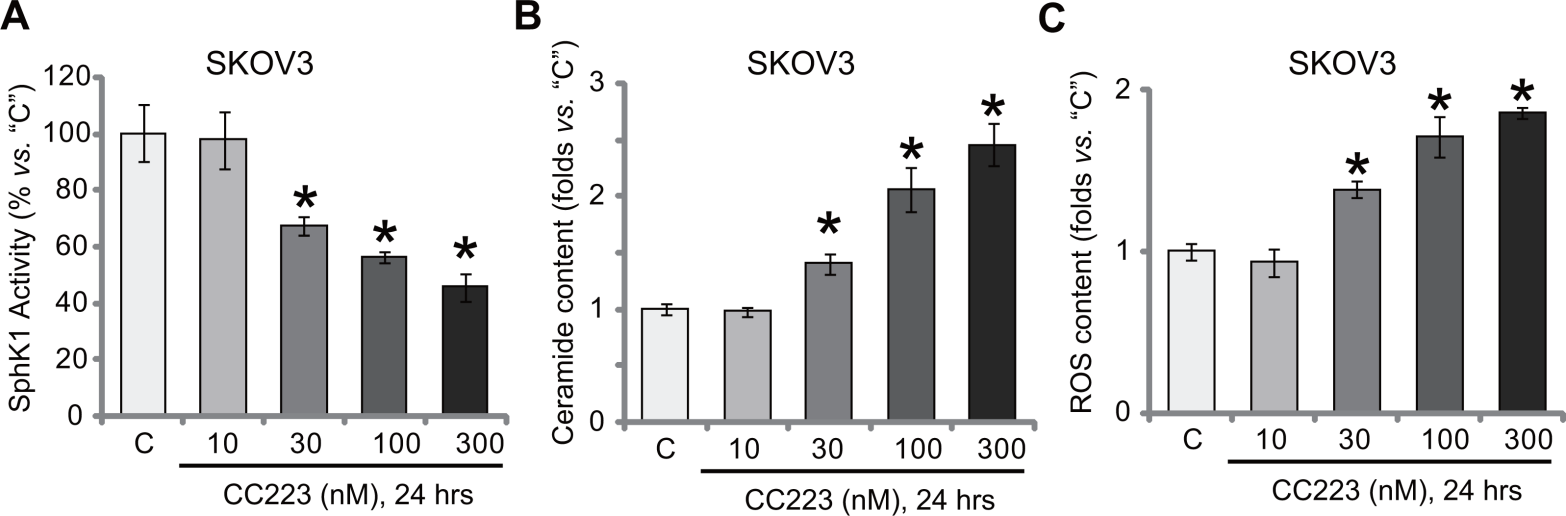
CC223 inhibits SphK1 and induces ROS production in SKOV3 cells SKOV3 cells were treated with applied concentration of CC223, cells were further cultured for 24 hours; Relative SphK1 activity (**A**), ceramide level (**B**) and ROS content (**C**) were tested by listed assays. Data were presented as mean ± standard deviation (SD, *n* = 5). *p < 0.05 *vs*. “C” group. Results in this figure were repeated three times, similar results were always obtained.

The above results suggested that other mechanisms beside mTOR inhibition could also be involved in CC223-mediated SKOV3 cell death. Growing evidences have suggested an pivotal function of sphingosine kinase 1 (SphK1) in ovarian cancer progression [21, 22, 35]. SphK1 is often over-expressed and/or sustained-activation in ovarian cancer cells, which is associated with cancer cell survival, proliferation and apoptosis-resistance [36, 37]. On the other hand, inhibition or silence of SphK1 could lead to ceramide production and cell apoptosis [36, 37]. We showed that SphK1 activity was also decreased following CC223 treatment in SKOV3 cells (Figure 5A). Consequently, cellular ceramide level was increased (Figure 5B). Intriguingly, our results also found that reactive oxygen species (ROS) content was also increased in CC223-treated SKOV3 cells (Figure 5C). Notably, CC223-induced SphK1 inhibition, ceramide production and oxidative stress were not changed by introduction of caAkt1 (Data not shown), suggesting that these actions by CC223 were independent of mTOR inhibition. Together, CC223 inhibits SphK1 and induces ROS production in ovarian cancer cells.

### CC223 inhibits SKOV3 tumor growth in nude mice

**Figure 6:**
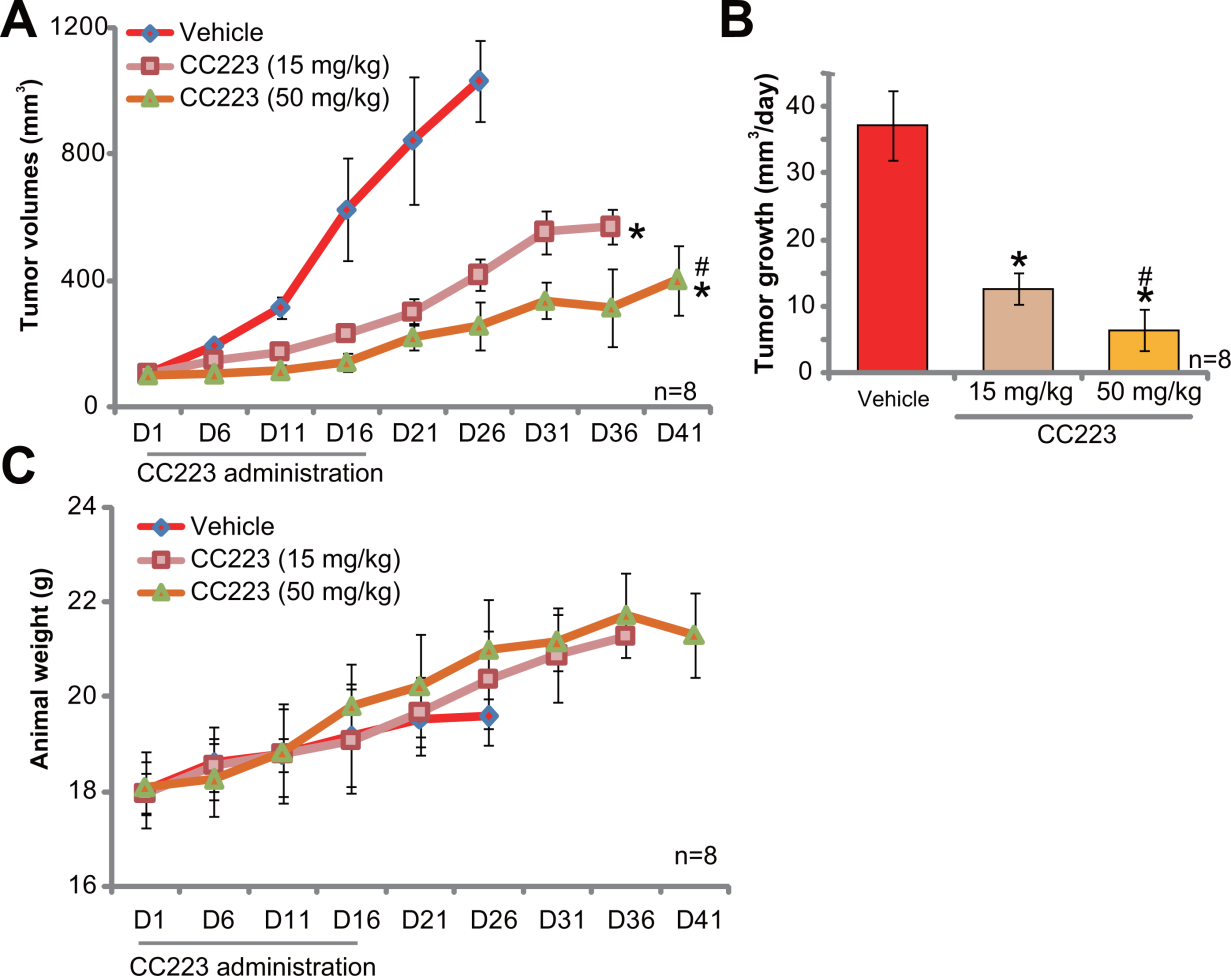
CC223 inhibits SKOV3 tumor growth in nude mice Randomly-assigned nude mice (*n* = 8 per group) bearing SKOV3 xenograft tumor were administrated (via gavage) with CC223 (15 or 50 mg/kg body weight, daily) or vehicle control (“Vehicle”) for 16 consecutive days; Tumor volume (**A**) and mice body weight (**C**) were recorded every 5 days for total 40 days; Estimated daily tumor growth (in mm^3^ per day) was also presented (**B**); *p < 0.05 *vs*. “Vehicle”. ^#^ *p* < 0.05 *vs*. CC223 at 15 mg/kg.

At last, the potential anti-ovarian cancer activity of CC223 *in vivo* was tested. SKOV3 cells were injected (*s.c*.) to the nude mice to establish xenograft tumors. The tumor-bearing mice were then treated with/out CC223. Tumor growth curve results in Figure 6A showed that oral administration of CC223 (15 or 50 mg/kg body weight, gavage) largely inhibited SKOV3 tumor growth in nude mice. CC223 at 50 mg/kg was more potent than 15 mg/kg in suppressing SKOV3 tumors (Figure 6A), showing a dose-dependent response *in vivo*. Meanwhile, estimated tumor growth, in mm^3^ per day, was also largely inhibited with CC223 administration (Figure 6B). Mice body weights, the indicator of mice general health, were not significantly changed by the above-mentioned CC223 treatments (Figure 6C). Experimental mice with CC223 treatment also didn't present with any signs of apparent toxicities, which is consistent with previous findings [18–20]. Collectively, these results confirm that CC223 oral administration inhibits SKOV3 tumor growth in nude mice.

## DISCUSSION

Ovarian cancer remains the major cause of gynecological cancer mortality, with over 14,000 women expected to die from the disease each year [3, 4]. mTOR signaling is frequently hyper-activated in ovarian cancer [14, 15]. Therefore, mTOR represents an attractive oncotarget for therapeutic interventions [14, 15]. Indeed, mTOR inhibitors are being tested in various stages of clinical development for ovarian cancer [14, 15].

Two mTOR complexes, mTORC1 and mTORC2, mediate mTOR signalings [8]. Rapamycin and its analogs (“rapalogs”) are mTORC1 inhibitors [38–41]. The anti-cancer activity of these rapalogs are overall weak or moderate [38, 40]. Rapalogs bind to FKBP12, leading to only partial inhibition of 4E-BP1 phosphorylation [38, 40]. Also, rapalogs could not directly inhibit mTORC2, which is also important for ovarian cancer tumorigenesis and progression [42–44]. Further, mTORC1 inhibition will often lead to activation of key oncogenic cascades. Specifically, it was shown that Akt and ERK-MAPK could be activated as feedback response by rapalogs [38, 40]. Also, the rapalogs’ solubility *in vivo* is not satisfactory as well [16, 45]. Therefore, recent research efforts have developed mTOR kinase inhibitors, which directly bind to the kinase domain of mTOR, leading to complete blockage of mTOR kinase activity. These inhibitors will then block both mTOR1 and mTORC2 [30, 46].

Intriguingly, our results showed that CC223 was even more potent in killing SKOV3 cells than the other mTOR kinase inhibitor AZD-2014 and the PI3K-Akt-mTOR pan inhibitor LY294002. Further, restoring mTOR activation by introduction of caAkt1 only partially inhibited CC223-induced killing of SKOV3 cells. These results indicated that mTOR-independent mechanisms should also be involved in CC223-mediated anti-SKOV3 activity. Indeed, our results discovered that CC223 inhibited SphK1 and induced ROS production in SKOV3 cells. Right now, the underlying mechanisms of SphK1 inhibition and ROS production by CC223 were still uncertain. Yet, it should be noted that restoring mTOR activation by caAkt1 failed to reinstate SphK1 activity or to inhibit ROS production. Meanwhile, AZD-2014 and LY294002 had no significant effect on SphK1 activity nor ROS production in SKOV3 cells (Data not shown). Therefore, SphK1 inhibition and ROS production shall be the unique actions of CC223. This could explain the superior activity of this mTOR kinase inhibitor against ovarian cancer cells *in vitro* and *in vivo*. Anyhow, the associated signaling mechanisms shall require further investigations.

## MATERIALS AND METHODS

### Chemicals and reagents

CC223 was purchased from MCE (Shanghai, China). LY294002, AZD-2014 and rapamycin were obtained from Selleck (Shanghai, China). Caspase inhibitors, z-VAD-fmk, z-DEVD-fmk and z-LEHD-fmk, were purchased from Calbiochem (Shanghai, China). Antibodies of this study were provided by Cell Signaling Tech (Shanghai, China).

### Culture of established cell lines

The two established ovarian cancer cell lines, SKOV3 and CaOV3, were provided by the Cell Band of Shanghai Institute of Biological Science (Shanghai, China). Cells were cultured in RPMI 1640/DMEM medium with 10% FBS. The normal ovarian epithelial cell line, IOSE-80, was provided by Dr. Fang [21]. IOSE-80 cells were cultured as described [21]. Cell culture reagents were purchased from Gibco BRL (Shanghai, China).

### Primary culture of human ovarian cancer cells

Three lines of primary ovarian cancer cells (“OC1”, “OC2” and “OC3”) were provided by Dr. Fang [21]. Primary cancer cells were maintained in 50% 199 medium and 50% 105 medium (Sigma), supplemented with 15% FBS, 4 ng/mL EGF (Sigma), 1000 U/mL penicillin, 100 μg/mL streptomycin, 10 mM HEPES, 100 nM nonessential amino acids and 1 mM sodium pyruvate. The protocols were approved by the Ethics Review Committee of all authors institutions, and were conducted according to the principles of Declaration of Helsinki.

### MTT assay of cell survival

Cells were seeded onto 96-well microtiter plates (5 × 10^3^ per well). After the indicated treatment, cell viability was tested via MTT dye, which measures *A*_490_ nm of the dissolved formazan product.

### Trypan blue staining assay

Cells with the CC223 treatment were stained with trypan blue dye, which only stays into the “dead” cells. Trypan blue percentage was recorded via an automatic cell counter.

### Colony formation assay

Ovarian cancer cells were maintained onto 60-mm culture plates at 1000 cells per plate. Cells were treated with CC223 for 7 days. Afterwards, the remain surviving colonies were stained with Coomassie Blue, and were counted manually.

### Cell cycle analysis

Following the CC223 treatment, ovarian cancer cells were fixed in 70% ethanol, and were stained with propidium iodide (PI). Beckman Coulter flow cytometer was then applied to FACS analysis of cell cycle. G1, S and G2M phase percentages were recorded [47, 48].

### BrdU incorporation assay

BrdU ELISA assay kit, purchased from the Cell Signaling Tech, was applied to test cell proliferation after CC223 treatment [49]. The ELISA OD value at 450 nM was recorded as the indicator of cell proliferation.

### Single-stranded DNA ELISA assay of cell apoptosis

With CC223 treatment, single-stranded DNA (ssDNA) apoptosis ELISA (Chemicon International, Temecula, CA) assay kit was utilized to quantify cell apoptosis. The detailed protocol was described in other studies [50, 51].

### Caspase activity assay

After CC223 treatment, 25 μg of cytosolic extracts (per condition) were mixed with caspase assay buffer [21] and indicated caspase substrate: Ac-DEVD-AFC for caspase-3, Ac-LEHD-AFC for caspase-9, or Ac-IETD-AFC for caspase-8. After incubation, the amount of liberated AFC was tested via a spectrofluorometer (Thermo) [21].

### TUNEL assay of apoptosis

Following CC223 treatment, cells were subjected to the TUNEL dye assay, which only stained the nuclei of apoptotic cells. Each assay analyzed at least 300 cells of same condition. TUNEL ratio (vs total nuclei) was recorded.

### Western blotting assay

Protein lysates (25 μg protein/sample) were subjected to SDS–polyacrylamide gel running, and were transferred onto PVDF membranes. The blots were immunoblotted with applied primary antibodies, which were then detected with HRP-conjugated secondary antibodies. Enhanced chemiluminescence (ECL) reagents were applied to show interested bands [52, 53].

### Co-Immunoprecipitation (Co-IP) assay

As reported previously [28], after CC223 treatment, 600 μg of protein lysates per condition were pre-cleared by incubation with protein A/G Sepharose. The pre-cleared lysate samples were then incubated with anti-mTOR antibody (Cell Signaling Tech) overnight. Protein A/G Sepharose (30 μL per treatment) was then added again to the lysates. mTOR complexes, captured by the A/G Sepharose, were washed and subjected to Western blotting assay.

### Constitutively active-Akt1 (caAkt1) cells

The stable SKOV3 cells (via puromycin selection), expressing constitutively active mutant Akt1 (caAkt1) or the empty vector (Ad-GFP), were gifts from Dr. Zhang [21]. Expression of caAkt1 was verified by Western blotting assay.

### Assay of SphK1 activity

SphK1 activity assay was described previously [21, 22]. Briefly, after the applied treatment, 25 μg cell lysates per condition were incubated with D-erythrosphingosine containing medium [21]. The reaction was terminated by HCl (1 N) and chloroform/methanol/HCl (100:200:1, v/v) buffer [21]. Afterwards, the solution were subjected to vigorous vortex, phases were separated. Radio-labeled sphingosine-1-phosphate (S1P) was separated by 60 thin-layer chromatography (TLC) on silica gel G60-plates with chloroform/acetone/methanol/acetic acid/water (10:4:3:2:1, v/v) as solvent. The phosphate incorporation was then quantified via a scintillation counter (LS-6500, Beckman, Shanghai, China) [54]. The SphK1 activity was valued as pmol/hour/g protein, and was always normalized to the control level.

### Ceramide content test

The intracellular ceramide content was analyzed by the previously described protocol [55], which was valued as fmol by nmol of phospholipid. Ceramide content in the CC223-treated cells was always normalized to that of untreated control group.

### Assay of cellular reactive oxygen species (ROS) content

After CC223 treatment, ROS level was measured through the DCFH-DA fluorescent dye (Invitrogen) assay. The detailed protocol was described previously [56, 57]. Briefly, cells were incubated with 10 μM of DCFH-DA for 30 min, and were analyzed for fluorescence via the Fluorescence Microplate Reader (Synergy 2, BioTek, Winooski, VT).

### SKOV3 xenograft assay

Female nude mice (5–6 weeks old, 17.5–18.5 grams) were injected *s.c*. with SKOV3 cells (5 × 10^6^ per mouse, in Matrigel). When tumors reached at around 100 mm^3^ in volume, mice were randomized into three groups, and daily treatment with vehicle, 10 mg/kg or 50 mg/kg of CC223 (*p.o*., daily, for 16 days). Tumor volume was calculated via the formula: Volume (mm^3^) = (*d*^2^ × *D*)/2, in which *d* and *D* were the shortest and the longest tumor diameter. All animal experiments were approved by Institutional Animal Care and Use Committee (IACUC) and NIH animal regulations.

### Statistical analysis

Data were analyzed by SPSS software (Version 17.0). ANOVA was performed for comparison across treatment regimes. Significance was set at *p* < 0.05 for all comparisons.
